# Simple Bone Cyst of the Mandible: Report of Two Cases

**Published:** 2011-03-30

**Authors:** M. Imanimoghaddam, A. Javadian Langaroody, S. Nemati, S. Ataei Azimi

**Affiliations:** 1 Associate Professor, Department of Oral and Maxillofacial Radiology, School of Dentistry and Dental Research Center, Mashhad University of Medical Sciences, Mashhad, Iran; 2 Assistant Professor, Department of Oral and Maxillofacial Radiology, School of Dentistry, Hamedan University of Medical Sciences, Hamedan, Iran; 3 Assistant Professor, Department of Oral and Maxillofacial Radiology, School of Dentistry, Guilan University of Medical Sciences, Guilan, Iran; 4 Postgraduate Student of Internal Medicine, Faculty of Medicine, Mashhad University of Medical Sciences, Ghaem Hospital, Mashhad, Iran

**Keywords:** Simple Bone Cyst, Mandibular Pseudocyst, Multilocular Lesion

## Abstract

Despite their names, simple bone cysts are no longer categorized as cysts since they lack an epithelial lining. However, their nature remains controversial. The internal structure is totally radiolucent, sometimes showing multilocular appearance, although the lesion does not contain true septa and the ridges of bone is produced by the scalloping effect. We presented two cases of histopathologically confirmed simple bone cyst. Radiographic features such as multilocular appearance and significant buccal and lingual expansion are not usual findings for simple bone cyst, whereas evident in our presented cases.

## Introduction

The pathogenesis of simple bone cyst (SBC) that does not derive from epithelium has not been completely explained or clarified; therefore, it has often been described as a pseudocyst within the bone. When the cavity is exposed surgically, it may be empty, or it may contain fluid. However, because it is lined with connective tissue, it is not a true cyst.[1]

The various names that have been associated with SBC are reflections of the possible etiologies of this lesion, including hemorrhagic bone cyst, traumatic bone cyst, progressive bone cavity and unicameral bone cyst.[2]

SBCs are seen more frequently in men than in women,[3][4] are usually found in young people, most commonly occurring in the second decade of life,[2][4][5] but the sex distribution is reported in some of literatures to be quite even.[6]

These lesions are most commonly located in the mandibular marrow space and above the inferior alveolar canal that extends posteriorly from the canine region.[2][3] Less often, they may occur in the incisor area of the mandible.[2]

Because these solitary bone cysts are often asymptomatic, most of them are discovered incidentally during examination of the teeth and that is the reason why they can become quite large.[2][7] Pain is the presenting symptom in some of the patients [3] and in some cases definite symptoms such as tooth sensitivity,[8] paresthesia,[2] fistulas,[8] and pathologic fracture of the mandible [4] have been reported. The involved teeth are vital and there is no mobility, displacement or resorption of their roots,[3][8] although resorption of the root in few of the articles has been reported.[5]

Radographically, the simple bone cyst is consistently radiolucent. It may seem multilocular occasionally in spite of not having septa which is due to the propensity of the lesion to scallop the endosteal surface of the outer cortex of the mandible.[2] The radiographic features such as true multilocular appearance and significant buccal and lingual expansion are not usual findings for the simple bone cyst. The differential diagnosis includes cyst-like lesions with a scalloping margin between the roots of the teeth and minimal expansion such as odontogenic myxoma and keratocystic odontogenic tumor. This article introduces two documented cases of simple bone cyst with some differences in the radiographic features.

## Case Presentation

The first case is a 10-year-old girl who was referred to the oral radiology department of the dental school of Mashhad university because of a bony hard swelling in the buccal vestibule of the anterior region of the mandible. Clinically, the overlying mucosa of the lesion was intact and there was pain and tenderness. Except for three of the teeth, no reaction was obtained with an electrical pulp tester in the involved teeth within the lesion, but the teeth turned to be vital at follow-up.

According to the statement of the patient’s parents, the swelling started developing from approximately 4 months ago and in the last 2 months its growth caused asymmetry in the left side of the chin. In the panoramic radiograph (ProMax; (Courtesy Planmeca Inc., Roselle, lL)), a well-defined radiolucent lesion extending from posterior to the mandibular right first molar to the distal aspect of the left first premolar was evident.

**Fig. 1:**
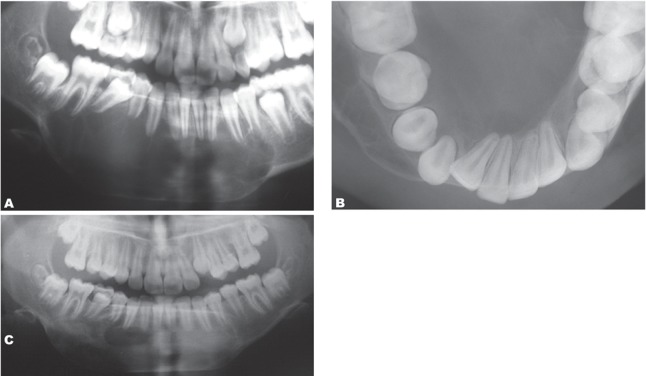
A 10-year-old girl presenting with painful swelling in the lower jaw.( A. Panoramic view shows a multilocular lesion in the symphysis region of the mandible. B. Occlusal projection shows considerable buccal and lingual expansion. Note the septa which are straight and at right angles to the periphery, mimicking central giant cell granuloma appearance. C. Follow-up panoramic X-ray taken one year after the surgery displays resolution of the lesion.)

The scallop outline in the superior border of the lesion and displacement of the right lateral and second premolar were evident. Lamina dura of some of the teeth was totally or partly destroyed. The internal structure of the lesion on panoramic view was as multilocular appearance that included wispy, ill-defined trabeculae and in some areas of the lesion there was evidence of fine, straight septa making a tennis racket-like or step-ladder like pattern (Fig. 1A). On occlusal projection, some of the trabeculae were at right angles to the periphery. In addition, the occlusal view revealed a considerable expansion of the buccal and especially lingual cortical plates (Fig. 1B). The differential diagnosis included central giant cell granuloma, aneurysmal bone cyst and odontogenic tumors (probably mural or unicystic ameloblastoma and odontogenic myxoma). The patient was operated after 30 days. During the operation an empty cavity without epithelial lining was exposed. Simple bone cyst was the diagnosis in surgical observation. A tissue sample was excavated from the cavity wall for histopathological examination. Histopathological study revealed only reactive changes with hemorrhage and loose vascular fibrous tissue adjacent to the bone. No epithelial lining was found. In the follow-up study approximately 3 months after operation, his chief complaint was swelling in the area of the right mandible, but there was no pain. A panoramic radiograph taken one year after the surgery revealed good osseous fill within the lesion (Fig. 1C).

The second case is a 22-year-old woman who was referred to our radiology department complaining of swelling on the right side of the mandible. On clinical examination, there was a hard bony expansion in the inferior border and buccal cortical plate of the mandible in the right premolar-molar region. Intraoral study revealed expansion of the cortical bone, either buccal and lingual. Four of the involved teeth on the right side showed no reaction to the pulp tester. In the panoramic radiograph (ProMax; (Courtesy Planmeca Inc, Roselle, lL)) a well-defined radiolucent lesion with a scalloping border between the roots of the teeth was revealed.

**Fig. 2:**
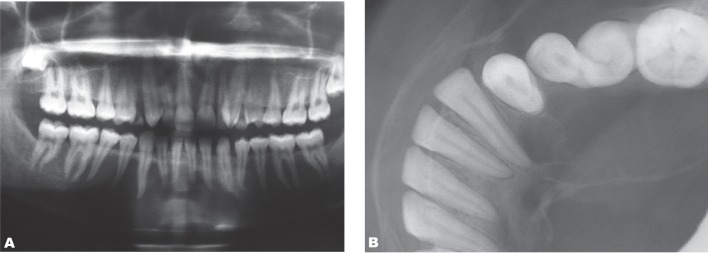
A 22-year-old woman complaining of severe swelling on the right side of the mandible.( A. Panoramic projection shows a radiolucent lesion with scalloping effect in the superior border. B. Occlusal view indicates buccal and lingual expansion. Note the double boundary expansion of the buccal cortical plate.)

The lesion extended from anterior to the mandibular right first molar to the left first premolar (Fig. 2A). On periapical view there was no evidence of root resorption or displacement of the teeth, but lamina dura in some teeth was partly destroyed. Occlusal projection revealed buccal and lingual expansion particularly on the right side of the mandible. There was evidence of an uneven or undulating expansion in the buccal cortical plate, which gave the appearance of a double boundary (Fig. 2B). The differential diagnosis was central giant cell granuloma and mural or unicystic ameloblastoma. On surgical exploration, there was a cavity containing some fluid. The histopathological study showed a cancellous bone cavity without an epithelial lining. Diagnosis established to be a traumatic bone cyst. On the follow-up study after six months, the patient complained of swelling in the same region, but on the panoramic view, the size of the lesion was decreased.

## Discussion

Simple bone cyst is not classified as a true cyst because the lesion lacks an epithelial lining. It is found to be empty or filled with liquid and/or connective tissue.[2] They are found mostly during the second decade of life,[2][4][5] though they can also be found in the older age population of over 40 years of age.[2] The sex distribution is reported to be quite even,[6] or men are affected somewhat more frequently,[3][4] a female predominance has also been reported.[5] Most SBCs are commonly painless lesions without signs and symptoms which are detected in the routine radiographic examination incidentally.[3][7] There might be swelling, but that occurs less commonly.[9] In both of our cases, there was a significant bony expansion associated with pain in the first patient. Pain or paresthesia would occur only when there is a close relation to the inferior dental nerve.[2] The involved teeth are usually vital.[3] Hancen et al.[9] reported that fewer than 15 percent of the patients had nonvital teeth involved with the lesion, but they believed that non-vitality is not a result of the lesion. In this study, some of the involved teeth within the lesion in both patients did not respond to the pulp test, but the teeth turned to be vital at follow-up. In addition, there was displacement of two teeth by the lesion in the first patient.

Location was predominantly in the premolar-molar area of the mandible.[2][4][10] In some instances, the lesions crossed the midline.[10] Maxillary involvement has also been reported.[10] In both of the mentioned cases, the lesion crossed the midline. The majority of these cysts are reported as single, distinct and isolated lesions of the mandible, although multiple lesions have been detected too.[11]

The outline scalloping between the roots of adjacent teeth is considered a characteristic feature of the lesion.[4][5][8][10] The margins may vary from smooth well-defined corticated to ill-defined.[5][10] Several internal characteristics such as presence of trabeculae,[8][10] complete radiolucency[2] and a cloudy appearance were identified.[10] An interesting aspect of the first case was its well-defined multilocular appearance that was revealed on radiological study. The multilocular cysts have been reported in some articles as cases causing misdiagnosis in preoperative study. They were considered radiologically as ameloblastoma or keratocystic odontogenic tumor,[8][10] whereas, in the presented cases, on the panoramic and occlusal view, there was evidence of straight septa as a tennis racket-like or step ladder-like pattern. On occlusal projection some of the trabeculae were at right angles to the periphery more characteristic for central giant cell granuloma and aneurysmal bone cyst. Occasionally, an expansion of the cortical plate, which is usually buccal is noted.[3] An additional important feature in the mentioned cases is considerable expansion of buccal and especially the lingual plate that was as undulating appearance in the second patient. This appearance is a characteristic finding for central giant cell granuloma. Although displacement of the involved teeth by the cyst has not been a common finding,[3][8] in some of the reports it has been documented.[8] Root resorption is rare and can cause disappearance of the lamina dura in some cases.[9] In both mentioned cases, lamina dura of some teeth were totally or partly destroyed, but there was no root resorption.

Although it is generally accepted that SBCs may undergo spontaneous resolution,[12] surgical exploration is necessary in order to establish the diagnosis [2][5] and is regarded as the curative procedure in the mandibular region.[5] The time between diagnosis and complete resolution of the lesion ranged from 2 years to 7 years, depending on the age of the patient at presentation. [5] However, in the report by Suei et al.,[13] in follow-up examination of 132 cases, greater than 20 percent of SBCs of the jaws recurred. Hence, clinical and radiological follow-up after surgery is strongly indicated.[6] Based on the results obtained in our study and other literatures, it may be concluded that SBCs are a radiological dilemma, since they could have different radiographic appearances either in the internal structure or effect the surrounding structures. We therefore suggest surgical exploration before any radical surgery.
